# Gastrointestinal Stromal Tumours (GIST) in Young Adult (18–40 Years) Patients: A Report from the Dutch GIST Registry

**DOI:** 10.3390/cancers12030730

**Published:** 2020-03-20

**Authors:** Nikki S. IJzerman, Cas Drabbe, Dide den Hollander, Mahmoud Mohammadi, Hester van Boven, Ingrid M. E. Desar, Hans Gelderblom, Dirk J. Grünhagen, An K. L. Reyners, Max M. van Noesel, Ron H. J. Mathijssen, Neeltje Steeghs, Winette T. A. van der Graaf

**Affiliations:** 1 Department of Medical Oncology, Netherlands Cancer Institute, Plesmanlaan 121, 1066 CX Amsterdam, The Netherlandscas.drabbe@student.ru.nl (C.D.); D.denHollander@radboudumc.nl (D.d.H.); n.steeghs@nki.nl (N.S.); 2 Department of Medical Oncology, Erasmus MC Cancer Institute, Erasmus University Medical Center, Doctor Molewaterplein 40, 3015 GD Rotterdam, The Netherlands; a.mathijssen@erasmusmc.nl; 3 Department of Medical Oncology, Radboud University Medical Center, Geert Grooteplein Zuid 10, 6525 GA Nijmegen, The Netherlands; Ingrid.Desar@radboudumc.nl; 4 Department of Medical Oncology, Leiden University Medical Center, Albinusdreef 2, 2300 RC Leiden, The Netherlands; M.Mohammadi@lumc.nl (M.M.);; 5 Department of Pathology, Netherlands Cancer Institute, Plesmanlaan 121, 1066 CX Amsterdam, The Netherlands; h.v.boven@nki.nl; 6 Department of Surgical Oncology, Erasmus MC Cancer Institute, Erasmus University Medical Center, Doctor Molewaterplein 40, 3015 GD Rotterdam, The Netherlands; d.grunhagen@erasmusmc.nl; 7 Department of Medical Oncology, University Medical Center Groningen, University of Groningen, Hanzeplein 1, 9713 GZ Groningen, The Netherlands; a.k.l.reyners@umcg.nl; 8 Department of Solid Tumors, Princess Maxima Center for Pediatric Oncology, Heidelberglaan 25, 3584 CS Utrecht, The Netherlands; M.m.vanNoesel@prinsesmaximacentrum.nl

**Keywords:** GIST, young-adult patients, mutations, treatment, outcome

## Abstract

Gastrointestinal stromal tumour (GIST) is a disease of older adults and is dominated by *KIT*/*PDGFR* mutations. In children, GIST is rare, predominantly occurs in girls, has a stomach location and generally lacks *KIT*/*PDGFR* mutations. For young adults (YA), aged 18 to 40 years, the typical phenotypic and genotypic patterns are unknown. We therefore aimed to describe the clinical, pathological and molecular characteristics of GIST in in YA. YA GIST patients registered in the Dutch GIST Registry (DGR) were included, and data were compared to those of older adults (OA). From 1010 patients in the DGR, 52 patients were YA (54% male). Main tumour locations were stomach (46%) and small intestine (46%). GIST genetic profiles were mutations in *KIT* (69%), *PDGFRA* (6%), SDH deficient (8%), NF1 associated (4%), *ETV6-NTRK3* gene fusion (2%) or wildtype (10%). Statistically significant differences were found between the OA and YA patients (localisation, syndromic and mutational status). YA presented more often than OA in an emergency setting (18% vs. 9%). The overall five-year survival rate was 85%. In conclusion, YA GISTs are not similar to typical adult GISTs and also differ from paediatric GISTs, as described in the literature. In this series, we found a relatively high percentage of small intestine GIST, emergency presentation, 25% non-*KIT/PDGFRA* mutations and a relatively good survival.

## 1. Introduction

Gastrointestinal stromal tumours (GISTs) are found throughout the gastrointestinal tract and arise from interstitial cells of Cajal [1]. Their estimated incidence is 10–15 per million [2]. GIST patients have a median age at diagnosis of 65 years, and less than 10% are diagnosed before the age of 40 [3,4,5].

The distinct presentation of paediatric GISTs is well described in the literature. While GISTs in adults arise in the stomach (40–60%) or in the small intestine (25–40%), in children, the stomach (90%) is the predominant site of origin, and multifocal GISTs are common [6,7]. Furthermore, in children, GISTs have a female predilection [8] and are more often associated with genetic syndromes, such as the Carney–Stratakis syndrome, Carney’s triad syndrome and Neurofibromatosis type 1 (NF1) [9,10,11]. 

Regarding mutational status, adult GISTs are commonly driven by activating mutations in either the *KIT* proto-oncogene (75%) or the *PDGFRA* (platelet-derived growth factor receptor A) oncogene (15%) [1,12,13], and 10% were formerly named wildtype (WT) for *KIT* and *PDGFRA* [14]. However, genetic research has unveiled other sporadic genetic alterations leading to GISTs, including RAS pathway gene mutations and mutations in the four succinate dehydrogenase (SDH) subunits [15,16,17]. Therefore, the description WT GIST is disappearing, and it is now common practice for oncologists to describe which genetic alterations patients have been tested for [18]. In contrast to the molecular characteristics of adults with GIST, children are *KIT* and *PDGFRA* WT in the vast majority (85–90%) [7,19,20,21,22]. Finally, metastases in adults are mostly located intra-abdominally [1,23], whereas, in children, lymph node and lung metastases are more common [24].

In addition to the classical paediatric and adult GIST presentation as defined above, a rare ‘paediatric-type’ presentation of GIST in (young) adults has been described in the literature, as well. Despite their adult age, these ‘paediatric-type’ GISTs are *KIT/PDGFR* WT and present with gastric lesions with multinodular or plexiform growth pattern and epithelioid or mixed cell histology. The ‘paediatric-type’ GIST predominantly affects women, often metastasizes to the lymph nodes and has a relatively indolent course of disease [25].

The young adult (YA) population has gained recognition in oncological research as a group with not only specific psychosocial needs but also distinct biological features [26,27,28]. The literature on both clinical and biological GIST characteristics in YA is scarce. This retrospective cohort study aims to describe the clinical, pathological and molecular characteristics of GIST in YA patients. 

## 2. Results

### 2.1. Patient Characteristics 

From 1010 patients registered in the Dutch GIST Registry (DGR) in March 2019, 52 YA patients (aged 18 to 40 years, 5%) were identified (Table 1). A small majority was male (54%), and the median age at diagnosis was 35 years (range 18–40). The main primary tumour locations were stomach (46%) and small intestine (ileum/jejunum/duodenum, 46%); median tumour size at diagnosis was 60 mm; and 87% had local disease at diagnosis. Two patients had multifocal GIST: one patient with a germline mutation (small bowel) and one patient without a known familial syndrome (stomach). Four YA patients had a known genetic predisposition for GIST, and three patients had a second malignancy (one patient had melanoma, one patient malignant peripheral neural sheath tumour (MPNST) and one patient also had breast and thyroid cancer). Most of the metastases that occurred were intra-abdominal/peritoneal or in the liver, with only one case of lymph node metastases. The most common symptom was active gastrointestinal bleeding (22%). In eight asymptomatic patients, the GIST was a coincidental finding. According to the Miettinen risk classification, 21 patients had at baseline a high-risk tumour (41%).

### 2.2. Pathology

The histology of the primary tumours was predominantly spindle cell (76%), and the baseline mitotic rate was low in 69% (Table 2). GIST genetic profiles were reported as *KIT* mutation 69%, *PDGFRA* mutation 6%, SDH deficient 8%, NF1 associated 4%, *ETV6-NTRK3* (translocation-Ets-leukaemia virus and neurotrophic tyrosine kinase, only tested in one of the WT patients) gene fusion 2%, quadruple WT 6%, triple WT 2% and *KIT/PDGFRA* WT 2%. The WT tumours were located in the stomach (4/5) and in the small intestine (1/5). Altogether, 25% of the patients had a non-*KIT/PDGFR* mutation. One patient had a double primary mutation: *KIT* exon 11 and *KIT* exon 9.

### 2.3. Treatment

**Figure 1:**
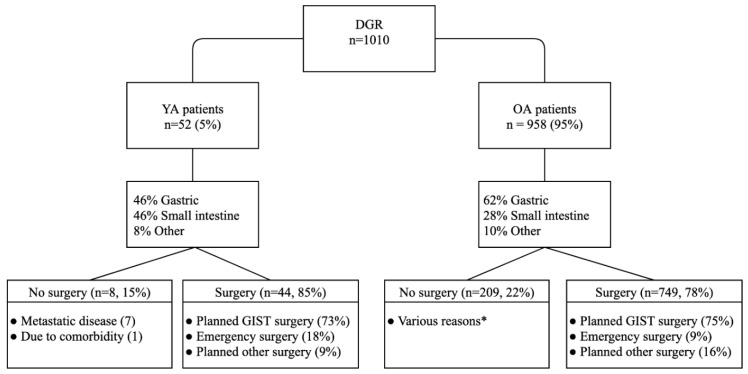
Study flowchart. * various reasons, such as metastatic disease, significant comorbidity, irresectable tumours or patients’ preference. DGR = Dutch GIST Registry; YA = young adult; OA = older adult.

Patients in Dutch GIST centres are generally treated according to standard guidelines. Of all YA patients in this cohort, 14 received neoadjuvant treatment (30%). Of the 45 patients with localized disease, three did not undergo surgery, as a result of progressive disease on neoadjuvant therapy (two) or comorbidities (one). Two patients received surgery of their primary tumour in the metastatic, emergency setting. In total, 44 patients underwent surgery of the primary tumour (85%) (Figure 1). In eight patients, emergency surgery was performed due to perforation or active bleeding (six small intestine; two gastric). The resection margins were microscopically free from tumour in 93%, and five patients (11%) had complications, with none requiring further surgery. Of the 44 surgically treated patients, seven patients received neoadjuvant and adjuvant treatment and 12 received adjuvant treatment only.

### 2.4. Outcome

With a median follow-up time of 52 months (95% Confidence Interval (CI): 31.5–72.5), the median overall survival (OS) for the YA patients was 8.9 years (95% CI: 7.4–10.4), with an estimated five-year survival rate of 85% (95% CI: 71.6–97.5). Of the 44 patients with resection of the primary tumour, 13 (30%) developed recurrence: two local, eight distant and three both local and distant recurrences. 

**Figure 2:**
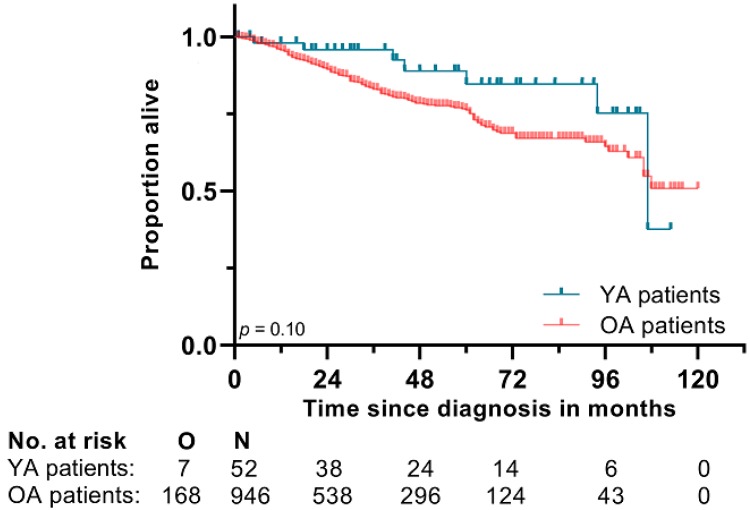
Overall survival curve of the young adult (YA) patients and older adult (OA) patients. O = number of observed events; N = number of patients at risk.

The median follow-up time in older adults (OA) was 37 months (95% CI: 33.0–41.0). The median OS time was not reached, but the estimated five-year survival rate was 76% (95% CI: 72.2–79.7). There was no significant difference in OS between the YA and OA patients (*p* = 0.10) (Figure 2).

### 2.5. Impact of GIST on Life 

In 56% of all patients, the psychosocial impact of their disease on life was both discussed by the oncologist and noted in the electronic patient record (EPR). This was 15% for the possible influence of the disease or treatment on fertility. The (in)ability to work/study was discussed and noted in 12 (46%) of the 26 patients receiving (neo) adjuvant therapy; seven patients (58%) had no limitations, four reported to be working/studying less than before diagnosis; and one could no longer continue work/study. For the 18 YA patients treated in the palliative setting, (in)ability to work/study was discussed and noted in eight (44%), of which one (13%) reported no limitations; five were (63%) studying/working less than before; and two (25%) stopped working/studying altogether. Eleven patients were referred to a clinical geneticist.

### 2.6. Comparison of Age Groups

#### 2.6.1. Comparison within the YA Cohort

**Figure 3:**
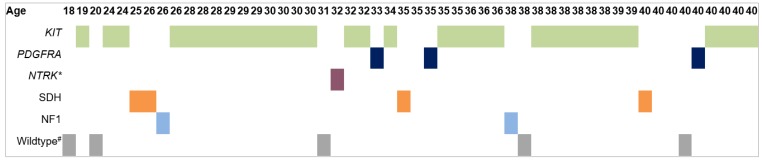
Distribution of mutation status among our young adult cohort *ETV6-NTRK3 gene fusion. # Wildtype for at least *KIT* and *PDGFR*.

Group 1 (age 18–29 years, median 26) consisted of 15 patients, and group 2 (30–40 years, median 38) of 37 patients. No significant differences in clinical and tumour characteristics were found between the two YA age subgroups (Table 3). Furthermore, no differences were found in OS (*p* = 0.81) nor in RFS (*p* = 0.75). Concerning the mutation distribution among the YA cohort, no clear turning point was found where more wildtypes in younger patients (as in paediatric population) shifted to more *KIT* mutated patients in older patients (as in adult population) (Figure 3).

#### 2.6.2. Comparison between the YA and OA Patients

The OA age group consisted of 958 patients (Table 4). Tumours were significantly more often localized in the small intestine in the YA age group, as compared to the OA age group (46% vs. 28%, *p* = 0.03). YA patients had a syndromic presentation more often than the OA age group (*p* = 0.047). Furthermore, YA patients significantly more often had non-*KIT/PDGFRA* positive tumours than patients in the OA group (25% vs. 11%, respectively; *p* = 0.008). Concerning treatment, YA patients tended to have surgery in an emergency setting more often than OA patients (18% vs. 9%, *p* = 0.06) (Figure 1). YA patients did not present more often with metastatic disease compared to OA (13.5% vs. 24.6%, *p* = 0.09). Furthermore, no significant differences were found between the two groups in gender, primary tumour size, risk classification, histology, baseline mitotic rate or the number of patients receiving surgery.

## 3. Discussion

This study shows that GIST at YA age is rare, representing only 5% of a large database. Paediatric GISTs have a female predilection and are rarely *KIT/PDGFRA* mutated [6]. With an almost equal gender distribution and increased presence of *KIT* or *PDGFRA* mutations, YA GISTs differ from paediatric GIST. However, YA GISTs are also not fully similar to the typical adult GISTs either, as they have a lower percentage of *KIT* and *PDGFRA* mutations (76% vs. 90%) and are more frequently located in the small intestine (46% vs. 28%). Within the YA age group, we could not demonstrate a clear turning point in age at which the mutational status shifted from WT to *KIT/PDGFRA* mutation, but that may be due to the still relatively small number of YA patients.

Comparison of the data from this study with age-related GIST data in the existing literature is challenging. This is due to the scarcity of GIST data in the YA population, the divergent composition of age-groups studied and the incomplete reporting on mutational status. Three studies reported on individual patients, and we used these data to construct a YA (18–29) and a paediatric age group (<18) [19,22,29]. Fero et al. studied a large AYA population (13–39) and an OA population (≥40); however, they did not report on mutational status [30] (Supplementary Table S1). The almost-equal gender distribution in the YA and OA cohort of this study is in accordance with the large AYA and OA population studied by Fero et al. [30]. Small intestine localisation seems to be less prevalent in the OA population compared with the YA population of this study, which is in line with the previous literature. Furthermore, this study confirmed that YA patients do not significantly more often present with metastatic disease compared to the OA population.

Concerning the mutational status, which was only scarcely reported before, our results were similar to the very small population (*n* = 30) of the combined studies of Prakash and Kang [19,29]. The amount of *KIT* and *PDGFRA* mutations does not seem to be as low as in paediatric patients, but also not as high as in OA patients. Since we found no clear turning point, there seems to be more evidence for a gliding scale, where an increase in age also increases the probability of a *KIT/PDGFRA* mutated GIST [31]. In two recent studies conducted in wildtype GIST patients, the reported median ages were 21 and 23 years, which supports the theory that most WT GISTs occur at a younger age [32,33].

In our cohort, YA GIST patients tended to undergo surgery more often in an emergency setting compared to OA. The relatively high percentage of small-intestine localisations might account for this [34]. Another possible explanation would be that the rarity of GIST at the YA age leads to a delay in (correct) diagnosis. Delay of diagnosis in sarcomas, especially at this age, remains a problem, and efforts to educate doctors and patients should be continued [35].

In over half of these young adults, the impact of the disease on life was not documented in their file and therefore possibly not discussed. For these YA patients who are diagnosed with cancer in the prime time of their lives, it is essential to take psychosocial factors (e.g., relationships, fertility, sexuality, financial toxicity and workability) into account. With a five-year survival of 85%, these survivorship issues are not trivial.

The five-year OS reported in this study is in line with the 82.4% five-year OS reported by Fero et al. in a large cohort of AYA patients aged 13–39, with GIST [30]. A study conducted in elderly (≥75 years) GIST patients reported a median OS of just 34 months [31]. The five-year survival rate of YA in our cohort seemed superior to the survival rate of OA (85% vs. 76%), but this was not significant. Notably, there was a difference in median follow-up, and the influence of other age-related factors, such as comorbidities, was not corrected for and could also explain (a part of) the difference in survival rate.

As a side note, several Dutch databases with paediatric cancer patients were searched for GIST, and over the same time period, only one paediatric patient was diagnosed. This illustrates how extremely rare paediatric GIST is.

For future research, it would be interesting to compare separate molecular subgroups between the YA and OA patients, in order to find out if the differences between the age groups persist. However, since GIST in YA patients is rare, and molecular subgroups are therefore extremely small, this research question can only be answered in an international setting, with an adequate sample size. Although the data was obtained from the DGR, a database containing information on GIST patients diagnosed for 10 years, the rarity of YA GIST makes that the numbers are still relatively small. Moreover, when the DGR was established, it was not common practice to test for *NTRK* fusion genes and *BCOR* gene mutations. Despite these limitations, this study on YA GIST is the largest series of a national population, including mutational status. Given its therapeutic implications, it is recommended to test from now on the quadruple WT GISTs for *NTRK* (neurotrophic tyrosine kinase) fusion genes as part of standard care.

## 4. Materials and Methods

### 4.1. Patient Selection and Variables

All patients who were 18 to 40 years old at diagnosis, who were diagnosed with GIST between January 2009 and January 2019 and who were registered in the Dutch GIST Registry (DGR) were included. The DGR is a prospectively maintained database, including patients ≥18 years with GIST treated in one of the five Dutch GIST centres (LUMC Leiden, Erasmus MC Rotterdam, UMC Groningen, Radboud UMC Nijmegen and the Netherlands Cancer Institute Amsterdam). Patient (age, gender, symptoms at diagnosis, presence of second malignancies and syndromic presentation), tumour (location, size, status at diagnosis (e.g., localized disease or metastatic disease), pathology (histology, mitotic rate and mutation status), treatment (surgery yes/no, surgery setting (e.g., emergency setting, planned operation for GIST and planned other surgery), (neo)adjuvant therapy yes/no) and follow-up data were obtained from the database. Furthermore, clinical, treatment and outcome data of all older adults (OA, >40 years old) were collected from the DGR, to compare data between OA and YA. Within the comparison of tumour location, locations with less than 100 patients in the OA group were classified as ‘other’. The DGR was approved by the local independent ethics committee (PTC14.0057). We adhered to the STROBE guidelines for cohort studies.

### 4.2. Pathology

GIST diagnosis was confirmed by positive immunohistochemistry (IHC) for CD117 and/or DOG1. Histology was described as spindle cell, epithelioid cell or mixed cell type. The required total count of mitoses is per 5 mm^2^ on the glass slide section. With the use of older model microscopes, 50 HPF was used which we considered semi-equivalent to 5 mm^2^. Low mitotic rate was defined as ≤ 5 mitoses/5 mm^2^ and high as >5 mitoses/5 mm^2^. GIST specific mutations tested by Next Generation Sequencing (NGS) were *KIT, PDGFRA* and *BRAF*. Patients without *KIT/PDGFRA/BRAF* mutations and where no SDH analysis was performed were considered triple wildtype (WT). Patients without *KIT/PDGFRA/BRAF* mutations and SDHB deficiency (considered deficient in the absence of SDHB protein expression on IHC [36]) were considered quadruple WT. Based on size, tumour site and mitotic activity, patients were subdivided into risk classification groups according to Miettinen’s criteria [37]. 

### 4.3. Quality of Life 

In this YA cohort, it was explored how often the psychosocial impact on life (e.g., depression, body image and relationships), influence on fertility and (in)ability to work was documented explicitly in the EPR by the medical oncologist. Whether or not this had been discussed with a patient was purely based on documentation in the EPR. Data on referral to a clinical geneticist were also collected.

### 4.4. Statistical Analyses and Outcome

Statistical analyses were performed by using SPSS Statistics (IBM Corporation, version 25.0, Armonk, NY, USA). Survival estimates were obtained, using the Kaplan–Meier method, and compared by the log-rank test. Overall survival (OS) was calculated from the date of diagnosis to the date of death or last follow-up. Follow-up time was estimated with the reversed Kaplan–Meier method. Two subgroups within the YA group were compared: 18–29 years vs. 30–40 years. The comparative analyses were done by using Fisher’s exact test for categorical variables or Mann–Whitney U test for numeric variables. 

## 5. Conclusions

YA with GIST are rare and differ from OA with GIST, but they also differ from paediatric GIST, as described in the literature. In this series, YA patients had a relatively high percentage of small intestine localisation and relatively often presented in the acute setting. Absence of both *KIT* and *PDGFRA* mutations was seen in 25% of YA patients. A relatively favourable five-year OS of 85% was observed.

## Figures and Tables

**Table 1:** Clinical and tumour characteristics of total young-adult (YA) age group.

Characteristic	Total (%)
**No. of YA Patients**	52 (100)
**Median age at Diagnosis (Range)**	35 years (18–40)
**Gender**	
Male	28 (53.8)
Female	24 (46.2)
**Median Baseline Tumour Size in mm (Range)**	60 (6–370)
**Location**	
Gastric	24 (46.2)
Small intestine	24 (46.2)
Rectum	3 (5.8)
Oesophageal–cardiac junction	1 (1.9)
**Syndromic Presentation**	
No Yes	39 (90.7) 4 (9.3)
Neurofibromatosis 1	2
Carney triad	1
*KIT* exon 11 germline mutation	1
Unknown	9
**Symptoms at Diagnosis** ^*^	
Active gastrointestinal bleeding	11 (22.4)
Abdominal pain	9 (18.4)
Weight loss	9 (18.4)
Mass related symptoms (e.g., reflux, palpable mass, distended abdomen)	7 (14.3)
Anaemia related symptoms	6 (12.2)
Acute abdomen	6 (12.2)
Nausea/vomiting	4 (8.2)
Change in bowel habits	3 (6.1)
Analysis vitamin B12 deficiency	1 (2.0)
None (GIST coincidental finding)	8 (16.3)
Unknown	3
**Second Malignancies**	
No	49 (94.2)
Yes	3 (5.8)
**Tumour Status at Diagnosis**	
Local disease	45 (86.5)
Metastasized	7 (13.5)
**Risk Classification**	
None Low	2 (3.9) 19 (37.3)
Moderate	9 (17.6)
High Unknown	21 (41.2) 1

^*^ Multiple symptoms at diagnosis possible. GIST = Gastrointestinal stromal tumour.

**Table 2:** Pathology and tumour genetic results of young-adult patients.

Characteristic	No. (%)
**Histology**	
Spindle cell	37 (75.5)
Epithelioid	3 (6.1)
Mixed type	9 (18.4)
Not reported	*3*
**Baseline Mitotic Rate**	
Low (≤ 5/5 mm^2^)	35 (68.6)
High (> 5/5 mm^2^)	16 (31.4)
Unknown	1
**Mutation Status**	
*KIT*	34 (69.4)
Exon 11 *	32
Exon 9	3
*PDGFRA*	3 (6.1)
D842V	2
Non-D842V	1
SDH deficiency	4 (8.2)
NF1 associated	2 (4.1)
*ETV6-NTRK3* gene fusion	1 (2.0)
*KIT/PDGFRA* WT (*BRAF* and/or SDH not performed)	1 (2.0)
Triple WT (SDH not performed)	1 (2.0)
Quadruple WT	3 (6.1)
No mutational analysis performed	3

* One patient had both an exon 11 and exon 9 mutation in the primary tumour. NF1 = neurofibromatosis type 1; WT = wildtype.

**Table 3:** Clinical and tumour characteristics within the young-adult age group.

Characteristic	18–29 Years (*n* = 15)	30–40 Years (*n* = 37)	*p*-Value
**Median Age at Diagnosis (Range)**	26 (18–29)	38 (30–40)	
**Gender**			0.07
Male	5 (33.3)	23 (62.2)	
Female	10 (66.6)	14 (37.8)	
**Median Baseline Tumour Size in mm (Range)**	45 (14–170)	60 (6–370)	0.23
**Primary Site Grouped**			0.48
Gastric	5 (33.3)	19 (51.4)	
Small intestine	9 (60.0)	15 (40.5)	
Other	1 (6.7)	3 (8.1)	
**Histology**			0.74
Spindle cell	12 (80.0)	24 (73.5)	
Epithelioid	0 (0.0)	3 (8.8)	
Mixed type	3 (20.0)	6 (17.6)	
Unknown	0	3	
**Tumour Status at Diagnosis**			1.00
Local disease	13 (87.7)	32 (86.5)	
Metastasized	2 (13.3)	5 (13.5)	
**Risk Category**			0.53
Low/moderate	7 (46.7)	23 (62.2)	
High	7 (53.3)	14 (37.8)	
Unknown	1	0	
**Mutation Status**			0.72
*KIT*	10 (66.7)	24 (70.6)	
*PDGFRA*	0 (0.0)	3 (8.8)	
SDH deficient	2 (13.3)	2 (5.9)	
NF1 associated	1 (6.7)	1 (2.9)	
*ETV6-NTRK3* gene fusion	0 (0.0)	1 (2.9)	
WT (min. *KIT* and *PDGFRA*) Unknown	2 (13.3) 0	3 (8.8) 3	
**Baseline Mitotic Rate**			0.32
Low (≤ 5/5 mm^2^)	8 (57.1)	27 (73.0)	
High (> 5/5 mm^2^) Unknown	6 (42.9) 1	10 (27.0) 0	
**Surgery**			0.41
Yes No	14 (93.3) 1 (6.7)	30 (81.1) 7 (18.9)	
**Patients who Received Surgery**	14 (93.3)	30 (81.1)	0.41
**Resection Margin**			0.26
Negative (R0)	12 (85.7)	27 (96.4)	
Positive (R1/R2)	2 (14.3)	1 (3.6)	
Unknown	0	2	
**Tumour Rupture**			1.00
No	11 (84.6)	24 (80.0)	
Yes	2 (15.4)	5 (20.0)	
Unknown	1	1	

NF1 = neurofibromatosis type 1; WT = wildtype.

**Table 4:** Clinical and tumour characteristics in the young adult (YA) and older adult (OA) age groups.

Characteristic	Total (%) *n* = 1010	YA (%) *n* = 52	OA (%) *n* = 958	*p*-Value
**Median Age at Diagnosis (range)**	64 (18–95)	35 (18–40)	65 (41–95)	
**Gender**				0.89
Male	533 (52.8)	28 (53.8)	505 (52.7)	
Female	477 (47.2)	24 (46.2)	453 (47.3)	
**Median Baseline Tumour Size in mm (Range)**	72 (1–1000)	60 (6–370)	73 (1–1000)	0.09
**Primary Site Grouped**				0.03 *
Gastric Small intestine Other	604 (60.7) 290 (29.1) 101 (10.2)	24 (46.2) 24 (46.2) 4 (7.7)	580 (61.5) 266 (28.2) 91 (10.3)	
**Syndromic Presentation**				0.047 *
No	799 (96.7)	39 (90.7)	760 (97.1)	
Yes	27 (3.3)	4 (9.3)	23 (2.9)	
Unknown	184	9	175	
**Tumour Status at Diagnosis**				0.09
Local disease	767 (76.0)	45 (86.5)	722 (75.4)	
Metastasized	242 (24.0)	7 (13.5)	235 (24.6)	
Unknown	1	0	1	
**Histology**				0.26
Spindle cell	739 (78.3)	37 (75.5)	702 (78.4)	
Epithelioid	94 (10.0)	3 (6.1)	91 (10.2)	
Mixed type	111 (11.8)	9 (18.4)	102 (11.4)	
Unknown	66	3	63	
**Baseline Mitotic Rate**				0.76
Low (≤5/5 mm^2^)	491 (66.4)	35 (68.6)	462 (66.2)	
High (>5/5 mm^2^)	252 (33.6)	16 (31.4)	236 (33.8)	
Unknown	261	1	260	
**Mutation Status**				0.001 *
*KIT*	630 (75.9)	34 (69.4)	596 (76.3)	
*PDGFRA*	106 (12.8)	3 (6.1)	103 (13.2)	
*BRAF*	1 (0.1)	0	1 (0.1)	
SDH deficiency	13 (1.6)	4 (8.2)	9 (1.2)	
NF1 associated	16 (1.9)	2 (4.1)	14 (1.8)	
*ETV6-NTRK3* gene fusion	1 (0.1)	1 (2.0)	0	
Wildtype (at least *KIT* and *PDGFRA*)	63 (7.6)	5 (10.2)	58 (7.4)	
Unknown	180	3	177	
***KIT/PDGFRA* vs. non-*KIT/PDGFRA***				0.008 *
*KIT/PDGFRA*	736 (88.7)	37 (75.5)	699 (89.5)	
Non-*KIT/PDGFRA*	94 (11.3)	12 (24.5)	82 (10.5)	
**Patients who Received Surgery**	793 (78.5)	44 (84.6)	749 (78.2)	0.30
**Surgery Setting**				0.06
Planned operation	712 (90.7)	36 (81.8)	676 (91.2)	
Emergency setting	73 (9.3)	8 (18.2)	65 (8.8)	
Unknown	8	0	8	

* *p*-value < 0.05 was considered significant; NF1 = neurofibromatosis type 1.

## Supplementary Materials

The following is available online at https://www.mdpi.com/2072-6694/12/3/730/s1. Table S1: Adolescents and young adults (AYA) age-related GIST data in the existing literature.
