# Supplementary material for: Gastrointestinal Stromal Tumours (GIST) in Young Adult (18–40 Years) Patients: A Report from the Dutch GIST Registry
Source: Cancers (Basel). 2020 Mar 20;12(3):730. doi: 10.3390/cancers12030730 (PMC7140070; doi:10.3390/cancers12030730)
Supplement: Supplementary file 1 [file cancers-12-00730-s001.pdf]

Table S1. Adolescents and young adults (AYA) age-related GIST data in existing literature.

	**Paediatric Patients**	**AYA Patients**	**Adult Patients**	
Age range	≤18	18–29	13–39	≥40
Studies	Prakash et al. (2005) n = 5<br>Benesch et al. (2011) n =16<br>Kang et al. (2013) n =2	Prakash et al. (2005) n =10<br>Kang et al. (2013) n =20	Fero et al. (2017)	Fero et al. (2017)
Total no. of patients	23 (100)	30 (100)	392 (100)	5373 (100)
Gender				
Male	5 (21.7)	9 (30.0)	207 (52.8)	2767 (51.5)
Female	18 (78.3)	21 (70.0)	185 (47.2)	2606 (48.5)
Localization				
Stomach	18 (78.3)	12 (40.0)	210 (53.6)	3048 (56.7)
Small intestine	3 (13.0)	16 (53.3)	139 (35.5)	1465 (27.3)
Other	2 (8.7)	2 (6.7)	43 (10.9)	860 (16)
Presentation				
Local disease	13 (56.5)	Not reported	212 (54.1)	2937 (54.7)
Metastasized	3 (13.0)		148 (37.7)	1992 (37.1)
Unknown	7 (30.4)		32 (8.2)	444 (8.3)
KIT mutation				
Present	1 (4.3)	19 (63.3)	Not reported	Not reported
Not present	12 (52.2)	10 (33.3)		
Unknown	10 (43.5)	1 (3.3)		
PDGFRA mutation				
Present	1 (4.3)	2 (6.7)	Not reported	Not reported
Not present	5 (21.7)	18 (60.0)		
Unknown	17 (73.9)	10 (33.3)		
Survival	Incomplete data	20 ANED<br>7 AWD<br>3 DOD	5-year OS<br>82.4%	5-year OS<br>67.1%

ANED = Alive with no evidence of disease; AWD = Alive with disease; DOD = Died of disease.
